# Umbilical Cord Mesenchymal Stem Cell-Derived Small Extracellular Vesicles Deliver miR-21 to Promote Corneal Epithelial Wound Healing through PTEN/PI3K/Akt Pathway

**DOI:** 10.1155/2022/1252557

**Published:** 2022-07-14

**Authors:** Xiaolong Liu, Xuran Li, Guangyuan Wu, Pengfei Qi, Yanyan Zhang, Zhiyu Liu, Xinyue Li, Yu Yu, Xiangmei Ye, Yang Li, Dongguang Yang, Yueqiu Teng, Ce Shi, Xin Jin, Sen Qi, Yuting Liu, Shudan Wang, Ying Liu, Fenglin Cao, Qingran Kong, Zhenkun Wang, Hong Zhang

**Affiliations:** ^1^Central Laboratory, First Affiliated Hospital, Harbin Medical University, Harbin, China; ^2^College of Life Science, Northeast Agricultural University, Harbin, China; ^3^Eye Hospital, First Affiliated Hospital, Harbin Medical University, Harbin, China; ^4^NHC Key Laboratory of Cell Transplantation, Harbin Medical University, Harbin, China; ^5^Department of Laboratory Diagnostics, First Affiliated Hospital, Harbin Medical University, Harbin, China; ^6^Department of Hematology, First Affiliated Hospital, Harbin Medical University, Harbin, China

## Abstract

**Objective:**

Rapid restoration of corneal epithelium integrity after injury is particularly important for preserving corneal transparency and vision. Mesenchymal stem cells (MSCs) can be taken into account as the promising regenerative therapeutics for improvement of wound healing processes based on the variety of the effective components. The extracellular vesicles form MSCs, especially exosomes, have been considered as important paracrine mediators though transferring microRNAs into recipient cell. This study investigated the mechanism of human umbilical cord MSC-derived small extracellular vesicles (HUMSC-sEVs) on corneal epithelial wound healing.

**Methods:**

HUMSC-sEVs were identified by transmission electron microscopy, nanoparticle tracking analysis, and Western blot. Corneal fluorescein staining and histological staining were evaluated in a corneal mechanical wound model. Changes in HCEC proliferation after HUMSC-sEVs or miR-21 mimic treatment were evaluated by CCK-8 and EdU assays, while migration was assessed by in vitro scratch wound assay. Full-length transcriptome sequencing was performed to identify the differentially expressed genes associated with HUMSC-sEVs treatment, followed by validation via real-time PCR and Western blot.

**Results:**

The sEVs derived from HUMSCs can significantly promote corneal epithelial cell proliferation, migration in vitro, and corneal epithelial wound healing in vivo. Similar effects were obtained after miR-21 transfection, while the beneficial effects of HUMSC-sEVs were partially negated by miR-21 knockdown. Results also show that the benefits are associated with decreased PTEN level and activated the PI3K/Akt signaling pathway in HCECs.

**Conclusion:**

HUMSC-sEVs could enhance the recovery of corneal epithelial wounds though restraining PTEN by transferring miR-21 and may represent a promising novel therapeutic agent for corneal wound repair.

## 1. Introduction

Superficial corneal lesions can heal rapidly, and without complication, however, delayed corneal epithelial healing can lead to subsequent corneal infections with further complications, such as corneal scarring, thinning, ulceration, and even perforation. According to the World Health Organization, it is estimated that corneal opacities, including corneal ulceration, are the fourth leading cause of blindness worldwide [1]. Although several therapies exist and an increasing number of novel approaches are emerging, treatment of severe corneal epithelial defect can still be quite challenging. Therefore, a topical treatment that aids in the management and accelerated closure of corneal wounds would help reduce the risk of infections and scarring and thus improve visual outcomes.

Mesenchymal stem cell- (MSC-)-based therapies participated in renovating the structure and function of damaged or diseased tissues [2]. However, poor engraftment and limited differentiation of transplanted MSCs suggest that their beneficial effects might not be associated with their differentiation and direct replenishment of damaged tissue parenchymal cells [3, 4]. Currently, emerging evidence has shown that the therapeutic effect of MSCs mainly relies on paracrine activities [5]. MSCs can release extracellular vesicles (EVs) containing bioactive molecules that affect cellular processes in neighboring cells. Therefore, it may be possible to avoid the limitations and complications of stem cell therapy in the eye by using MSC-derived EVs as biomimetic agents to accelerate corneal wound healing [6].

EVs, specifically exosomes—a specific subpopulation of sEVs which originate from the multivesicular endosomes, are functional paracrine units of stem cells and have therapeutic effects similar to their parent cells, suggesting that they may provide a promising, cell-free therapeutic options [7–9]. The application of MSC-derived EVs alone can exert similar functions of MSCs and participate in the regulation of immune response, inflammatory disease, and wound healing [10]. The cellular bilayer lipid membrane of EVs protected proteins, mRNAs, and noncoding RNAs (ncRNAs) from destruction, which can be transferred to recipient cells for cell-to-cell communication [11]. ncRNA is considered as key posttranscriptional modulators of gene expression and can be transferred in active form via EVs to regulate the activity of certain cells. Among them, microRNAs (miRNAs) have emerged as the most important modulator [12]. miRNAs are a class of evolutionally conserved, single-stranded ncRNAs, which are either transcribed by RNA polymerase II from independent genes or introns of protein-coding genes [13]. miRNAs are crucial players during normal development, homeostasis, and disease, which participate in almost every biological process such as cell proliferation and survival [14].

MSCs from cord tissues are easily attainable and more primitive than MSCs isolated from adult sources. Previous studies have shown that human umbilical cord mesenchymal stem cell-derived small extracellular vesicles (HUMSC-sEVs) could transfer miRNAs and attenuate cell death and enhance cutaneous wound healing [15, 16]. Considering the similar wound healing process between skin and corneal, we studied the functions of HUMSC-sEVs in corneal wound repair. The present study demonstrated the therapeutic effect of HUMSC-sEVs using a corneal mechanical wound model. To investigate the mechanism underlying HUMSC-sEV-mediated corneal wound repair, we studied the effects of HUMSC-sEVs on human corneal epithelial cell (HCEC) migration and proliferation. Through high-throughput sequencing and bioinformatic analysis, we identified that miR-21 is carried by HUMSC-sEVs as crucial element contributing to HCECs' migration and proliferation by downregulating PTEN expression.

## 2. Materials and Methods

### 2.1. Primary Cell Culture and Characterization

The umbilical cord was obtained from the Department of Obstetrics and Gynecology of the First Affiliated Hospital of Harbin Medical University after harvesting informed consent for research purposes, which was approved by the Ethics Committee (ethical approval number: 201859). The collected umbilical cord Wharton's jelly tissue was cut into small pieces and then allowed to stick to the bottom of the cell culture plates. Dulbecco's Modified Eagle's Medium (DMEM) Low Glucose with 10% fetal bovine serum (FBS) and 100 U/mL penicillin-streptomycin (Gibco, USA) was added to the cells. The dissociated cells were washed with PBS and stained with antibodies CD90, CD105, CD73, CD34, CD11b, CD19, CD45, and HLA-DR using the BD™ Human MSC Analysis Kit. The FACS analysis was performed using a FACS Calibur™ flow cytometer (BD Biosciences), and the data were analyzed using the FlowJo software (BD Biosciences). The release of exosomes from HUMSCs was blocked by preincubating HUMSCs with 20 *μ*M GW4869 (Sigma, USA) for 24 hours. All cells used in our experiments were from early passages 3 to 5.

To avoid the influence of FBS-derived EVs on HUMSC-sEVs, HUMSCs used for sEV extraction were cultured using EV-free FBS which were centrifuged at 120,000 × g at 4°C for 18 hours using a Beckman Optima L-100 XP ultracentrifuge with a SW 32 Ti rotor.

### 2.2. Isolation and Identification of HUMSC-sEVs

For accuracy and clarity, we use the term “EVs” or “sEVs” instead of “exosomes” as small HUMSC-derived membranous structures following the International Society for Extracellular Vesicles (ISEV) recommended guidelines on EV nomenclature, isolation, and characterization [17, 18]. HUMSC supernatants were collected at different times every 24-48 hours and centrifuged at 300 × g for 10 minutes to pellet dead cells, and cell debris were removed by centrifuging at 2,000 × g for 10 minutes and then 10,000 × g for 30 minutes to eliminate large vesicles, and after centrifuging at 120,000 × g for 70 minutes, HUMSC-sEV pellets were washed with PBS and ultracentrifuged at 120,000 × g for another 70 minutes. All centrifugation steps were performed at 4°C. The purified sEVs were resuspended in PBS and stored at -80°C.

The concentration of HUMSC-sEVs was determined by BCA protein assay kit, as suggested by the manufacturer (Beyotime Institute of Biotechnology, China). The morphology of HUMSC-sEVs was observed by transmission electron microscopy (TEM) (JEOL JEM-1220, Japan). And the size distribution of HUMSC-sEVs was measured by nanoparticle tracking analysis (NTA, Malvern Zetasizer, England). The membrane protein markers (CD9, CD81, and CD63) were analyzed using Western blot.

To obtain the miR-21 knockdown HUMSC-sEVs, we transfected MSCs with miR-21 inhibitors (RiboBio, China) or negative control (NC) using Opti-MEM (Gibco, USA) and Lipofectamine Stem Transfection Reagent (Invitrogen) according to the manufacturer's instructions. After 48 hours of culture incubation, sEVs were isolated from culture supernatants by differential centrifugation as described above.

### 2.3. HCEC Culture and Transfection

The human corneal epithelial cell lines (HCEC, Bnbio, China) were cultured in DMEM High Glucose supplemented with 10% FBS (Gibco, USA). HCECs were seeded into 6-well or 12-well plates the day before treatment. Prior to HUMSC-sEVs or PBS treatment, HCECs were starved in serum-free DMEM for 24 hours at 50% confluence. HCECs were transfected with miR-21 mimics or miR-21 inhibitors and corresponding NC, pCDNA3.1-3×Flag-PTEN, and empty vector plasmid as indicated.

### 2.4. sEV Uptake Assay

For the sEV uptake analysis, 20 *μ*g purified HUMSC-sEVs in 100 *μ*l PBS were incubated with 1 *μ*M Dil (Beyotime Institute of Biotechnology, China) in the dark for 30 minutes, washed twice with PBS, ultracentrifuged at 120,000 × g for 70 minutes to remove nonbinding dye, and then resuspended in serum-free medium. HCECs were labeled with 5 mM CFSE in the dark for 20 minutes and washed twice with complete medium. 200 *μ*l cell suspension (5 × 10^4^/ml) was seeded into 35 mm glass bottom dishes (Cellvis, USA) and let the cells adhere to the glass for 12 hours. CFSE-labeled HCECs were cocultured with Dil-labeled HUMSC-sEVs for 2 hours. After washing with PBS and fixing in 4% paraformaldehyde, cell nuclei were stained with DAPI (Beyotime Institute of Biotechnology, China). Images were taken under confocal microscope (Zeiss LSM 710, Germany) and analyzed with supplementary software.

For TEM observation, HCECs were cocultured with HUMSC-sEVs (40 *μ*g/ml) for 2 hours and then fixed with 2.5% glutaraldehyde and postfixed with 3% osmium tetroxide (OsO4) for 2 hours. The specimen was dehydrated in a graded series of ethanol, embedded in EPON resin, and then imaged with TEM at 100 kV (Hitachi H-7650, Japan).

### 2.5. In Vitro Wound Healing Assay

HCECs were seeded into six-well plates and grown to confluence. The monolayer was scratched using a 200 *μ*l pipette tip and washed with serum-free medium to remove detached cells. Then, the cells were kept in coculture with HUMSC-sEVs or not. At different times, images of wound scratch were taken under a microscope. The scratch closure was analyzed by the ImageJ software. The percentage of wound closure was calculated as follows: migration area (%) = (*A*_0_ − *A*_*n*_)/*A*_0_ × 100, where *A*_0_ represents the initial wound area and *A*_*n*_ represents the wound area at the time of measurement.

### 2.6. In Vitro Cell Proliferation Assay

HCEC proliferation was measured using the cell counting kit-8 (CCK-8, Sigma, USA) according to the manufacturer's protocol. The optical density (OD) at 450 nm was measured with averages from three replicates using a microplate reader (BioTek Instruments, USA).

### 2.7. In Vitro EdU Proliferation Assay

Cell proliferation was also assessed using EdU Cell Proliferation Assay kit (RiboBio, China) according to the manufacturer's protocol. Briefly, after treatment, HCECs were exposed to 50 *μ*M 5-ethynyl-2′-deoxyuridine (EdU, RiboBio) for 2 hours at 37°C, and then, the cells were fixed in 4% paraformaldehyde. After permeabilization with 0.5% Triton-X100, the cells were reacted with 1× Apollo reaction cocktail for 30 minutes. Subsequently, the DNA contents of the cells were stained with Hoechst33342 for 30 minutes. Finally, the proportion of the cells incorporating EdU was determined with fluorescence microscopy (OLYMPUS, IX51).

### 2.8. Western Blot

HUMSC-sEVs and HCECs were lysed in lysis buffer containing protease and phosphatase inhibitor (Beyotime Institute of Biotechnology, China). Proteins were separated by electrophoresis after loading onto polyacrylamide gel and then transferred to the PVDF that was incubated with primary antibodies against phospho-Akt (4060s, CST), PTEN (9188, CST), CD9 (ab92726, Abcam), CD61 (ab59479, Abcam), and CD81 (00679767, Invitrogen) overnight at 4°C after blocking with 5% nonfat milk, followed by incubation with horseradish peroxidase- (HRP-) conjugated secondary antibody. Proteins were detected with a Western blot analysis system.

### 2.9. PCR

Total RNA of cells was extracted using TRIzol kit (Invitrogen, USA). RT-qPCR was carried out using the SYBR® Premix Ex TaqTM kit (Takara Bio, Japan) according to the manufacturer's instructions. The thermocycling conditions (Bio-Rad, CFX96) used were as follows: 95°C for 3 minutes, followed by 40 cycles of 95°C for 5 seconds, 60°C for 30 seconds, and 72°C for 30 seconds, and followed by a final extension of 72°C for 5 minutes. Relative expression of these genes was calculated by the 2^−*ΔΔ*Ct^ method.

### 2.10. Bioinformatic Analysis

HUMSC-sEV miRNA expression microarray GSE69909 ws downloaded from the GEO database. Target Scan, mirBase, and miRDB were used to predict the target genes of miRNAs enriched in sEVs. All the predicted targets have target prediction scores ≥ 80 were subjected to gene ontology (GO) analysis to investigate the underlying mechanism of the potential HUMSC-sEV miRNA and the target mRNAs during corneal reepithelialization.

### 2.11. Full-Length Transcriptome Sequencing

HCECs (2.5 × 10^6^ cells) were treated with 40 *μ*g/ml HUMSC-sEVs for 48 hours, and the same volume of PBS was added as control with three biological replicates. Total RNA was isolated using the TRIzol reagent according to the manufacturer's instructions. 1 *μ*g total RNA was prepared for cDNA libraries using cDNA-PCR Sequencing Kit (SQK-PCS109) protocol provided by Oxford Nanopore Technologies (ONT). Briefly, the template switching activity of reverse transcriptase enriches for full-length cDNAs and adds defined PCR adapters directly to both ends of the first-strand cDNA. And it follows cDNA PCR for 14 circles with LongAmp Tag (NEB). The PCR products were then subjected to ONT adaptor ligation using T4 DNA ligase (NEB). Agencourt XP beads was used for DNA purification according to ONT protocol. The final cDNA libraries were added to FLO-MIN109 flowcells and run on PromethION platform at Biomarker Technology Company (Beijing, China). The KOBAS software was used to test the statistical enrichment of differential expression transcripts in KEGG pathways.

### 2.12. Corneal Mechanical Wound Model and Treatment

The experimental protocols were approved by the Ethics Committee of First Affiliated Hospital of Harbin Medical University (ethical approval number: 2020100). Male Sprague-Dawley rats weighted 160–180 g (6-8 weeks old) were purchased from the animal experiment center of the Second Affiliated Hospital of Harbin Medical University. The rats were anesthetized with intraperitoneal injection and applied topically 0.5% proparacaine, and the corneal epithelium was removed up to the corneal/limbal border with AlgerBrush II (The Alger Company, Lago Vista, TX, USA) as previously described [19]. A unilateral corneal injury was created. Protocols were approved by the Harbin Medical University Animal Care and Use Committee guideline.

Rats were randomly divided into four groups and subconjunctival injected with 100 *μ*l PBS containing 2 × 10^6^ HUMSCs, equal amount of HUMSCs pretreated with GW4869, 40 *μ*g HUMSC-sEVs, or an equal volume of PBS, respectively. Wound residual area was monitored every 12 hours using fluorescein staining and photographed using a camera equipped Nikon FS-2 slit lamp biomicroscope. The percentages of residual defect were analyzed by the ImageJ software.

### 2.13. Histological Analysis

The eyes were enucleated and postfixed with 4% paraformaldehyde within 10 minutes after euthanasia. 4 *μ*m paraffin-embedded sections stained with hematoxylin and eosin (H&E) were used to observe the corneal structure and degree of corneal reepithelialization. The sections were photographed under light microscope (Olympus, Japan).

### 2.14. Statistical Analysis

All statistical analyses were performed using the Prism software. Data are summarized as mean ± standard deviation (SD). Student's *t-*test was used to determine statistically significant differences between samples. When multiple comparison analyses were required, statistical significance was evaluated by one-way ANOVA. All *P* values < 0.05 were considered statistically significant.

## 3. Results

### 3.1. Identification of HUMSCs and HUMSC-sEVs

**Figure 1:**
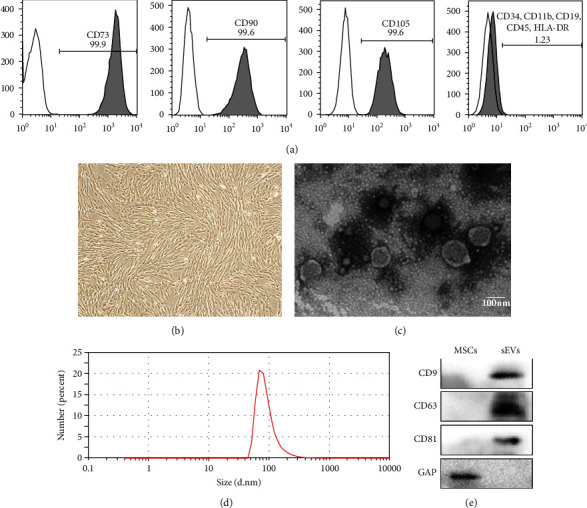
Morphological observation and identification of HUMSCs and HUMSC-sEVs. (a) Flow cytometry analysis of surface markers in HUMSCs. (b) Light morphology image of HUMSCs. (c) Morphology of HUMSC-sEVs under TEM. Scale bar, 100 nm. (d) Peak size of HUMSC-sEVs was around 80 nm as showed by NTA. (e) HUMSC-sEVs were positive for CD9, CD81, and CD63 as indicated by Western blot.

Results of flow cytometry analysis confirmed the presence of positive expressions of typical MSC markers CD105, CD90, and CD73, while the surface markers of hematopoietic cells such as CD34, CD11b, CD19, CD45, and HLA-DR were fairly weak to detect compared with the isotype control (Figure 1(a)). In addition, according to inverted microscopic observation, the morphology of the cells was regular long spindle with directional arrangement and presented a typical spindle shape, which grew as whirlpool or cluster (Figure 1(b)).

The classical structures of the isolated sEVs, including “rim of a cup” and double-layer membrane morphology, were observed by TEM (Figure 1(c)). NTA results demonstrated that the diameters of the particles were around 50–150 nm (Figure 1(d)). Protein markers of EVs were further confirmed by Western blot, including CD9, CD63, and CD81 (Figure 1(e)).

### 3.2. Application of HUMSCs or HUMSC-sEVs Promotes Corneal Wound Healing in a Rat Model

**Figure 2:**
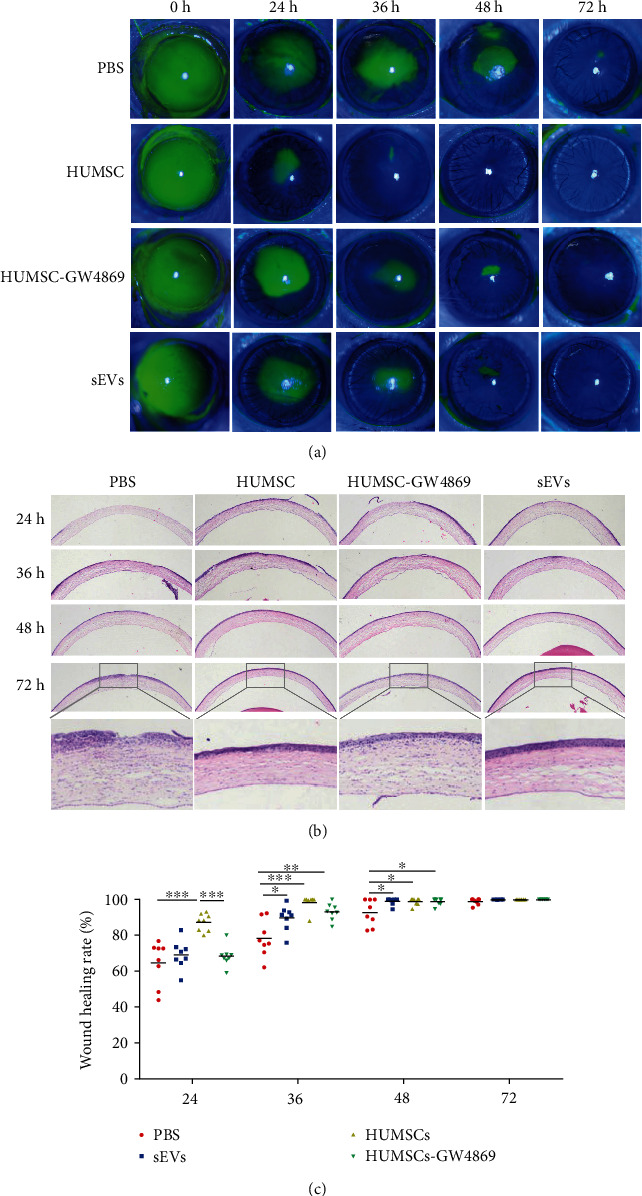
The effect of HUMSCs and HUMSC-sEVs on corneal epithelial wound healing in vivo. (a, c) Fluorescein-stained images of defect corneas, before and after treatment with HUMSCs, HUMSCs-GW4869, HUMSC-sEVs, or PBS. (b) H&E staining showed the histologic appearance of the cornea. Data are expressed as the means ± SD. ^∗^*P* < 0.05, ^∗∗^*P* < 0.01, and ^∗∗∗^*P* < 0.001, *n* = 8.

We found that subconjunctival injection of HUMSCs or HUMSC-sEVs can effectively promote the healing of corneal defects in rats, while inhibition of exosome secretion by GW4869 can attenuate HUMSCs mediated benefits at 24 hours (Figures 2(a) and 2(c)). The injured corneas treated with HUMSC and HUMSC-sEVs regained more regular arrangement and compact structure than those treated with PBS through assessing corneal tissue microstructure by H&E staining (Figure 2(b)).

## 4. HUMSC-sEVs Promote the Proliferation and Migration of HCECs In Vitro

**Figure 3:**
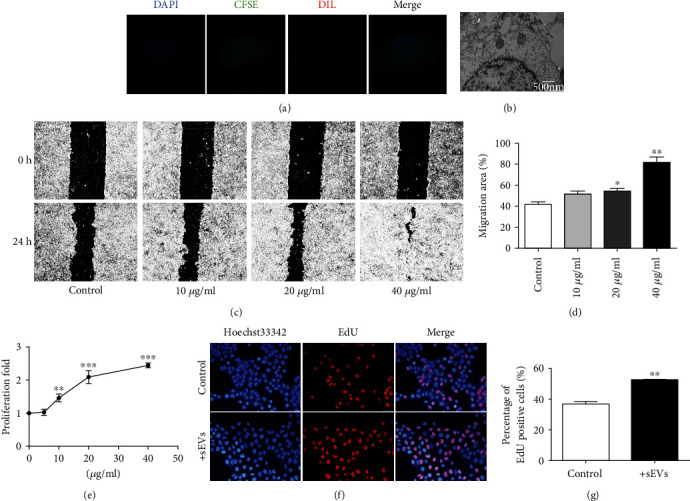
The effect of HUMSC-sEVs on HCECs' proliferation and migration in vitro. (a) Fluorescence images of CFSE-labeled HCECs (green) incubated with Dil-labeled HUMSC-sEVs (red). Nuclei were stained with DAPI (blue). (b) TEM of HCECs incubated with HUMSC-sEVs. (c, d) Representative images from in vitro scratch wound healing assays demonstrating that cell migrates into the cell-free region is significantly promoted in the presence of HUMSC-sEVs when compared to controls, *n* = 4. (e) CCK-8 assay showed increased proliferation of HCECs incubated with HUMSC-sEVs after 48 hours, *n* = 5. (f, g) The proliferating HCECs were detected by EdU incorporation. The cells were treated with HUMSC-sEVs or blank control, *n* = 3. Blue: nuclear staining (Hoechst33342); red: EdU staining. Data are expressed as the means ± SD. ^∗^*P* < 0.05, ^∗∗^*P* < 0.01, and ^∗∗∗^*P* < 0.001.

To demonstrate the uptake of sEVs, CFSE-labeled HCECs were cocultured with Dil-labeled sEVs and then visualized with laser scanning confocal microscope. The red nanoparticles represent the labeled sEVs that occurred in smaller clusters and were observed either surrounding the cell membrane or within the cytoplasm in HCECs. Localization results showed that sEVs derived from HUMSCs had been taken up by HCECs with the dye distributing within in the cell (Figure 3(a)). In addition, the fusion process was also observed by TEM (Figure 3(b)).

In order to evaluate whether HUMSC-sEVs stimulate HCEC migration, the effect on wound closure rates was investigated. The disparity of the remaining area during scratch wound assays confirmed the promigratory effects of HUMSC-sEVs with a dose-related trend after 18 hours of incubation (Figures 3(c) and 3(d)). Considering corneal healing is a dynamic interwoven process composed of cell proliferation, migration, and adhesion, and the proliferation ability is the basis. We further investigated whether HUMSC-sEVs could enhance the proliferation-promoting behavior of HCECs in vitro. The CCK-8 assay showed that the proliferation of HCECs after incubating with HUMSC-sEVs was significantly improved in a dose-dependent manner (Figure 3(e)). And the EdU assays for visualization of proliferating cells also demonstrated that HUMSC-sEV treatment increased the percentage of proliferating cells compared to controls (Figures 3(f) and 3(g)).

## 5. HUMSC-sEVs Promote HCECs' Proliferation and Migration through PI3K/Akt Pathway

**Figure 4:**
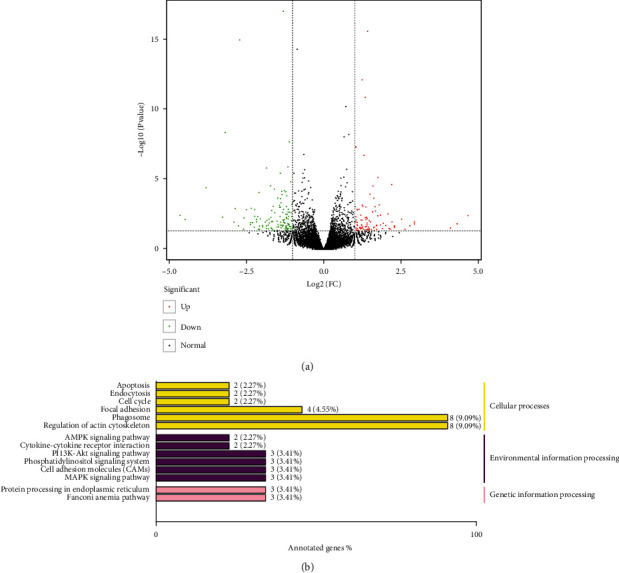
Transcriptome and pathway analysis of HUMSC-sEV treatment. (a) Volcano plot of DEGs between HUMSC-sEV-treated HCECs and HUMSC-sEV-untreated HCECs. Dots in green stand for downregulated DEGs, red dots mean upregulated DEGs, and black dots are nonsignificant DEGs. (b) The KEGG annotation results of the DEGs were classified according to the pathway types in KEGG. DEG: differentially expressed gene; KEGG: Kyoto Encyclopedia of Genes and Genomes.

To further investigate the potential mechanism of HUMSC-sEVs regulated proliferation and migration in HCECs, full-length transcriptome sequencing was used to detect the mRNA expression levels of related genes. 240 differentially expressed genes (DEGs) were identified (fold change ≥ 2 and *P* value < 0.05), including 104 upregulated DEGs and 136 downregulated DEGs (Figure 4(a)). Then, we interpreted the potential biological functions of DEGs from the gene function and signaling pathway through KEGG enrichment analysis and revealed that the PI3K/Akt signaling pathway, the phosphatidylinositol signaling system, cell adhesion molecules, and MAPK signaling pathway had a significant difference between before and after HUMSC-sEV-treated HCECs (Figure 4(b)). Previous studies demonstrated that PI3K/Akt pathway involved deeply in the modulation of the process of corneal epithelial wound healing [20]. Therefore, the PI3K/Akt signaling pathways involved in HCECs' proliferation and migration process after HUMSC-sEV treatment were explored.

## 6. miR-21 Regulates the PTEN/PI3K/Akt Signaling Pathway

**Figure 5:**
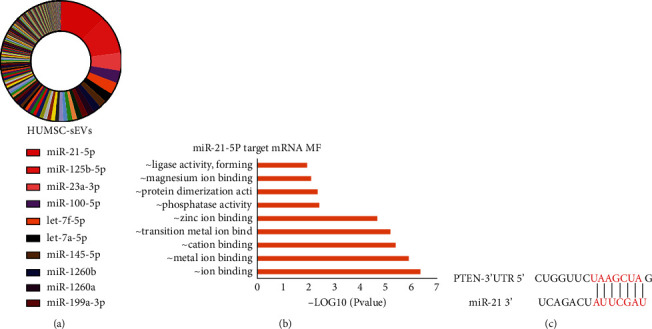
Identification of miRNAs contained in HUMSC-sEVs. (a) miRNA abundance analysis of HUMSC-sEVs. (b) mRNA targets for the miRNAs significantly enriched in HUMSC-sEVs were identified and GO analysis. (c) The binding site between miR-21 and PTEN mRNA. MF: molecular function; GO: gene ontology.

sEVs regulate a large number of physiological activities via miRNAs [21]. As miRNAs are abundant in HUMSC-sEVs, we hypothesized that HUMSC-sEVs promote the healing of corneal epithelial defect mainly through miRNAs. The downloaded dataset was used to determine the content of various miRNAs in HUMSC-sEVs [22]. Among the several miRNAs selectively enriched in HUMSC-sEVs, we focused on the most abundant one, miR-21 (Figure 5(a)). The downstream targets were predicted by Target Scan, mirBase, and miRDB databases and then imputed DAVID online to conduct GO analysis. The results showed that miR-21 was involved in the regulation of various molecular functions, containing calcium ion binding, peptidase inhibitor activity, growth factor activity, etc. (Figure 5(b)). Among them, the phosphatase activity may involve in the regulation of PI3K/Akt. miRNAs can exert their functions by interacting with the 3′ untranslated region (3′ UTR) or protein coding sequence of target mRNAs. According to the miRbase database, PTEN might be the potential downstream of miR-21 (Figure 5(c)).

**Figure 6:**
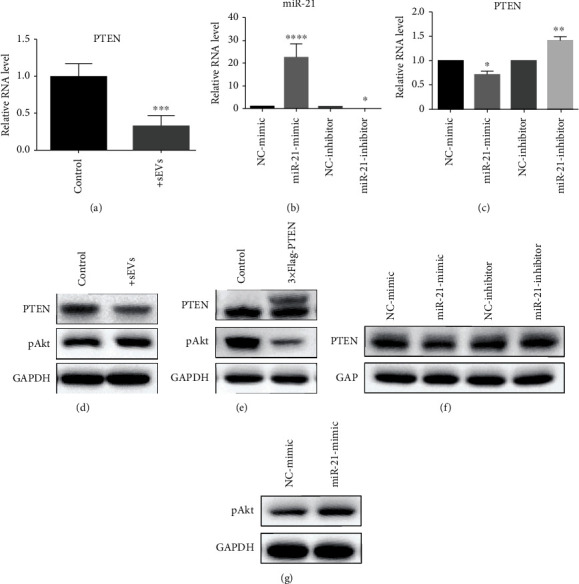
miR-21 regulates HCECs' proliferation and migration by activating PI3K/Akt pathway through targeting PTEN. (a, d) HUMSC-sEV treatment decreased the RNA and protein levels of PTEN in HCECs. (b) The expression level of miR-21 in HCECs. (c, f) The PTEN changed with miR-21 variation. (e) The expression of phospho-Akt after overexpression of PTEN. (g) The expression level of phospho-Akt after transfected with miR-21 mimics was detected by Western blot. Data are expressed as the means ± SD. ^∗^*P* < 0.05, ^∗∗^*P* < 0.01, and ^∗∗∗^*P* < 0.001.

Unsurprisingly, we found that reduced mRNA and protein expression levels of PTEN were identified within HCECs after treated with HUMSC-sEVs (Figures 6(a) and 6(d)). The activation of the PI3K/Akt pathway in HCECs following HUMSC-sEV stimulation was verified by assessing the phosphorylated Akt levels (Figure 6(d)). To confirm whether PTEN is a target of miR-21 in HCECs, we further measured the expression of PTEN in HCECs transfected independently with miR-21 mimics or inhibitors and their corresponding NC to verify the interaction between the miR-21 and PTEN by qRT–PCR and Western blot. Once transfected with miR-21 mimics, the protein levels of PTEN were significantly reduced in HCECs (Figure 6(f)), and the difference was also detected in transcription level (Figures 6(b) and 6(c)). We also found that the effects of miR-21 on the Akt phosphorylation were stimulative (Figure 6(g)). Meanwhile, overexpression of PTEN downregulated the phosphorylated Akt levels, which was important for proliferation and migration (Figure 6(e)). These results suggested that miR-21 regulates PTEN within HCECs via posttranscriptional modification.

## 7. HUMSC-sEVs Promote HCECs' Proliferation and Migration through miR-21

**Figure 7:**
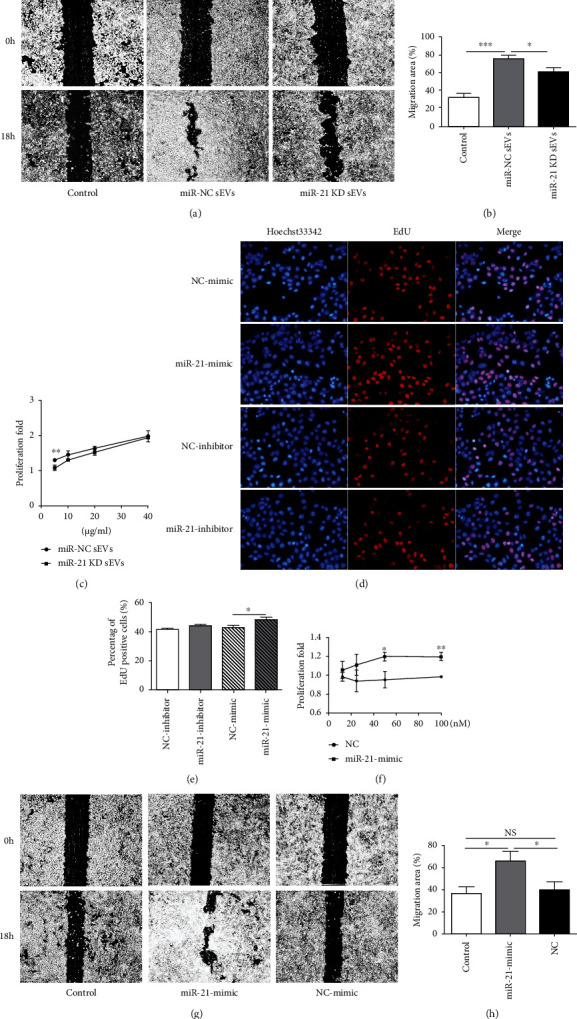
sEV-mediated transfer of miR-21 promotes HCEC proliferation and migration. (a, b) HCECs were treated with miR-21 KD HUMSC-sEVs or miR-21 contained HUMSC-sEVs for 18 h. The scratch assay showed the healing of the miR-21 KD HUMSC-sEV-treated group was slower than the miR-21 contained HUMSC-sEV-treated group, *n* = 5. (c) The CCK-8 assay showed the proliferation of the miR-21 KD HUMSC-sEV-treated group was lower than the miR-21 contained HUMSC-sEV-treated group after 18 hours, *n* = 3. (d, e) The proliferation of HCECs was detected by EdU incorporation after transfected with miR-21 mimics (at final concentration of 50 nM). Blue: nuclear staining (Hoechst33342); red: EdU staining, *n* = 3. (f) The CCK-8 assay showed the proliferation of the miR-21 mimic group was higher than control group after 48 hours, *n* = 3. (g, h) The scratch assay showed significantly faster wound closure in HCECs incubated with miR-21 mimics than NC after 18 hours, *n* = 5. Data are expressed as the means ± SD. ^∗^*P* < 0.05, ^∗∗^*P* < 0.01, and ^∗∗∗^*P* < 0.001. KD: knockdown.

In order to assess whether the sEV-mediated miR-21 transfer plays a role in HCECs' proliferation and migration, a subsequent knockdown experiment was conducted. HUMSCs were transfected with miR-21 inhibitors (at final concentration of 100 nM) or NC, and the culture supernatants were collected subsequently for isolating the sEVs. Then, HCECs were incubated with the same concentration of miR-21 contained or miR-21 knockdown HUMSC-sEVs for migration and CCK-8 analysis. Results showed that the upregulation of migration (Figures 7(a) and 7(b)), as well as proliferation (Figure 7(c)) induced by HUMSC-sEVs, was partially negated by miR-21 knockdown.

To further study the potential involvement of miR-21, HCECs were transiently transfected with miR-21 mimics or NC. Proliferation of HCECs following transfection with miR-21 mimics or NC was assessed using CCK-8 and EdU assay. miR-21 mimic transfection significantly promoted the proliferation of HCECs compared with the NC group (Figures 7(d)–7(f)). In addition, the ability of HCECs transfected with miR-21 mimics to regain monolayer integrity was raised compared with NC-transfected cells (Figures 7(g) and 7(h)).

Taken together, our data indicate that miR-21 in sEVs promotes HCECs' proliferation and migration by activating PI3K/Akt signaling pathway, which might play a critical role to enhance corneal epithelial wound healing.

## 8. Schematic Diagram

**Figure 8:**
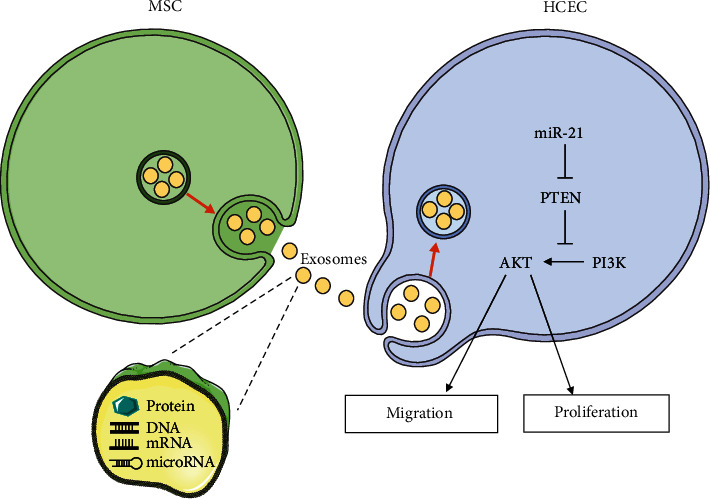
Schematic diagram describes the mechanism of HUMSC-sEVs in corneal epithelial defect.

We first determined the positive effects of sEVs derived from HUMSCs in promoting corneal epithelial wound healing and then determined the main molecules PI3K/Akt in the wound healing process and the fact that miR-21 was the most abundantly contained in HUCMSC-sEVs through bioinformatic analysis. Finally, we anchored PTEN as the downstream target of miR-21, which was the key link between miR-21 and related protein and determined that PTEN/PI3K/Akt were involved in cell proliferation and migration (Figure 8).

## 9. Discussion

Corneal epithelial damage is one of the most common ocular disorders, and novel treatments are needed to improve clinical outcomes for this type of disease. The current study demonstrated the beneficial effect of HUMSC-sEVs on corneal injury. To elucidate the potential mechanism associated with this activity, our in vitro results revealed that HUMSC-sEVs promote HCECs' proliferation and migration via repression of PTEN expression and downstream effects involving the phosphorylation of Akt. Moreover, miR-21 as an important regulator also showed the effect in promoting HCECs' proliferation and migration by targeting PTEN. Our results suggested that HUMSC-sEVs may be an exceptionally meaningful and promising approach for the healing of corneal defects.

As a cell-based therapy for treating human diseases has gained increasing interest over the last few decades, hundreds of clinics or clinical trials using human MSCs have carried out and showed that the application of MSCs can enhance wound healing [23] and ameliorate fibrosis [24]. In this study, we have shown that subconjunctival injection of HUMSCs facilitated corneal epithelial wound healing. However, several recent studies suggest that the therapeutic effects of MSCs may be largely mediated by paracrine effects involving proteins/peptides, hormones, and vesicles packaging various molecules [25]. Among such effectors, exosomes are considered to be the key effectors to exert therapeutic function. In order to further confirm if HUMSC paracrine effects dominate the process of cornea wound healing, HUMSCs were pretreated with GW4869, an exosome generation blocker [26]. Expectedly, HUMSCs were less effective in enhancing wound healing when the release of exosomes was blocked. These results implicating the function of HUMSCs particularly rely on exosome release into the microenvironment.

Researches have shown that independent application of MSC-derived sEVs can also play a critical role in promoting the repair of damaged tissues [27]. Moreover, MSC-derived have many advantages over MSCs, such as less safety concerns [28], long-term preservation and easy transportation [29], lower immunogenicity [30], and capacity to cross biological barriers [31]. Previous studies have shown that sEVs from corneal MSCs can reduce scar formation and increase the transparency of corneal healing [32]. sEVs from placental MSCs can reduce the inflammatory response during corneal alkali burn and promote the restoration of normal corneal structure [33]. sEVs from bone marrow MSCs promoted survival of retinal ganglion cells and regeneration of their axons through miRNA-dependent mechanisms [34]. In present study, for the first time, we proved that HUMSC-sEVs could promote the repair of corneal epithelium integrity and the healing process of corneal injury both in vitro and in vivo.

Since miRNAs were first identified by Lee et al., new miRNAs are still being discovered with the development of high-throughput sequencing technologies and computational and bioinformatic prediction methods [35]. Increasing evidences indicated that miRNAs can prevent target mRNA from translating into protein as posttranscriptional regulation [22]. In most cases, miRNAs interact with the 3′ UTR of target mRNAs in a complementary manner to suppress protein translation and then regulate cell proliferation, differentiation, development, and senescence [36, 37]. Acting as the crucial mediators of MSC-derived sEVs, miRNAs can provide sustained therapeutic effect and fundamental alterations of the local microenvironment, making it an ideal therapeutic biomolecule [38]. Many studies have validated the role of miRNAs in sEVs in various types of cells [39, 40]. In order to further explore how HUMSC-sEVs affect the corneal epithelial cells, we consulted GEO dataset and combined with bioinformatic analysis methods to analyze the content composition of HUMSC-sEV-derived miRNAs, and transcriptome sequencing was performed to identify the DEGs in HUMSC-sEV-treated HCECs compared to untreated condition. We found that sEVs derived from HUMSCs were rich in miR-21, which might act as the physiological and pathological regulatory factor. In our study, the sEVs extracted from miR-21 KD HUMSCs weaken the effect on HCECs' proliferation and migration compared with those extracted form miR-21 contained HUMSCs, implicating the function of HUMSC-sEVs partly depends on miR-21. miR-21 overexpression has the similar effect on promoting proliferation and migration of corneal epithelial cells. These results showed that miR-21 has a fundamental function on corneal epithelial cell amplification. miR-21 is a proliferation-related miRNA, and its role in wound healing was demonstrated in skin wound models [41] and cornea wound healing [42]. Despite previews results proved miR-21/SPRY2 axis participated in modulating epithelial phenotypes, promoted the migration of corneal epithelial cells, and enhanced the wound healing process, the mechanisms underlying miR-21 effect on corneal epithelial wound healing remain largely unknown.

PI3K/Akt pathway is a signal transduction pathway closely related to cell growth and proliferation and plays an important mediating role in proliferation, differentiation, and apoptosis of normal cells. The signal protein activity was increased in the tissue cells with strong proliferation ability [43]. Studies have shown that the activation of PI3K and Akt can trigger and accelerate the transformation and proliferation of skin epithelial cells, while the use of inhibitors can inhibit the proliferation of cancer cells and improve the level of programmed cell death [44, 45]. Once the PI3K/Akt signaling pathway was suppressed, corneal epithelial migration was delayed [46–48]. These observations from various experiments suggest that PI3K/Akt signaling may have the stimulatory effect in the maintenance of the corneal epithelium integrity. In our experiment, PI3K/Akt pathways were activated in HCECs' proliferation and migration promoted by HUMSC-sEVs. miR-21 could weaken the expression level of PTEN and increase PI3K/Akt signaling activation in HCECs.

The downstream of miR-21 has been verified based on the starBase database prediction, dual-luciferase reporter gene assay, and evidences from other researches [49–51]. PTEN was the potential effector, which belongs to tumor suppressor gene and inhibits the phosphorylation level of key proteins in various signaling pathways to play a negative function by promoting cell apoptosis and cell cycle arrest and regulating cell migration and other links [52]. Recent studies have shown that PTEN is involved in the pathological mechanism of myocardial injury and neurocognition, also in regulating corneal epithelial defects [48, 53–55]. In addition, PTEN remains the main negative regulator of PI3K/Akt signaling through its phosphoinositide phosphatase activity [56]. To confirm the relationship among miR-21, PTEN, and PI3K/Akt, we transferred miR-21mimics into HCECs and found that miR-21 overexpression could downregulate the expression level of PTEN, and this downregulation further induced the upregulation of phospho-Akt. These results demonstrated that miR-21 promoted HCECs' proliferation and migration by regulating PI3K/Akt via PTEN.

Although miR-21 is one of the most abundant miRNAs in HUMSC-sEVs, the regulation of cell migration and proliferation induced by HUMSC-sEVs was partially negated by miR-21 knockdown. miR-21 KD HUMSC-sEVs retain most of their biological activities suggesting that there are still other unidentified bioactive components. It is reported that EVs carry only low numbers of miRNA that are too few to elicit any biological responses [57, 58], and MSC-derived EVs are likely to play functional roles because of their proteins rather than RNAs [59]. Although we substantiated that miR-21 in HUMSC-derived-sEVs mediated the effect of proliferation and migration in HCECs, there still remains other cargoes (especially protein) function as similar roles await further investigations. In addition, as the situation of patient with severe corneal epithelial defect is much more complex than that in animal models, whether HUMSC-sEVs can promote the healing of severe corneal injury in clinical practice remains unknown.

However, sEVs are nanosized vesicles which could be delivered using a needle as small as possible, and their biological activity would not be affected by the increased inner pressure of the needle. We proposed that HUMSC-sEVs can not only be used as a local drug to promote corneal epithelial defects but also can be injected for more intraocular diseases that cannot be treated locally, thus serving as a putative therapeutic agent. Our study proved that the administration of HUMSC-sEV eye is a promising strategy for the treatment of corneal epithelial defect, which serves as a foundation for the development of more effective strategy in corneal wound healing.

## 10. Conclusions

In conclusion, this study firstly revealed the function of HUMSC-sEVs in promoting corneal epithelial cell proliferation and migration via upregulating the PI3K/Akt signaling pathway though restraining PTEN by transferring miR-21, leading to better corneal wound repair and regeneration. Our results offer a novel therapeutic agent for the treatment of a corneal wound as a cell-free therapy.

## Data Availability

The data that support the findings of this study are available from the corresponding author upon reasonable request.
